# Callous–Unemotional Traits and Intelligence in Children with Externalizing Behavioral Problems

**DOI:** 10.3390/children9111768

**Published:** 2022-11-17

**Authors:** Pamela Fantozzi, Pietro Muratori, Valentina Levantini, Irene Mammarella, Gabriele Masi, Annarita Milone, Alessia Petrucci, Federica Ricci, Annalisa Tacchi, Chiara Cristofani, Elena Valente

**Affiliations:** 1 IRCCS Fondazione Stella Maris, 56128 Pisa, Italy; 2 Department of Languages and Literatures, Communication, Education and Society, University of Udine, 33100 Udine, Italy; 3 Department of Developmental and Social Psychology, University of Padova, 35122 Padua, Italy

**Keywords:** verbal abilities, callous–unemotional, conduct problems, verbal comprehension, ADHD, oppositional defiant disorder, conduct disorder

## Abstract

Research on the association between callous–unemotional (CU) traits and intelligence yielded contradictory results. Moreover, several previous studies focused on global intelligence scores or verbal vs. nonverbal/performance abilities usually evaluated with short/abbreviated instruments. The current study builds on these previous works and explores the link between CU traits and intelligence using the full version of the Wechsler Intelligence Scale for Children—4th Edition (WISC-IV), which provides four different verbal and nonverbal abilities scores. This guarantees a more detailed evaluation of children’s intelligence and its relation to CU traits. The sample included children (*N* = 149; age 6–14 years old) with severe behavioral problems. Clinicians administered the WISC-IV, and parents completed questionnaires evaluating the child’s externalizing problems and CU traits. Findings showed that CU traits were associated with lower verbal comprehension scores after also controlling for gender, age, externalizing problems, and the other WISC-IV indexes. In addition, CU traits and externalizing problems did not interact in predicting the WISC-IV indexes, and there were no significant differences in the WISC-IV indexes between children with CU traits and high vs. low externalizing problems. The current study suggests the relevance of assessing and addressing verbal abilities in children with behavioral problems and CU traits.

## 1. Introduction

Externalizing problems are widely prevalent and are among the main reasons for a childhood referral to mental health and educational services. A research review estimated a worldwide-pooled prevalence of externalizing problems ranging from 4% to 8%, with a mean of 5.7% [1]. Similar results emerge from Italian epidemiological studies. For instance, Gritti et al. [2] found that 8.5% (3.85% borderline, 4.7% clinical) of children suffered from externalizing behavioral problems. These difficulties lead to several adverse outcomes and may represent a significant economic burden for the child, their family, their victims, and, more broadly, for society [3,4], with estimated public costs per child exceeding USD 70,000 over seven years.

Externalizing problems is an umbrella concept that encompasses a wealth of different behaviors (e.g., aggression, oppositional and defiant behavior) [5], and the presence of callous–unemotional (CU) traits seems to delineate a clinically relevant and etiologically distinct subgroup of youths with severe externalizing problems. CU traits in children are characterized by a lack of guilt and remorse, shallow affect, and reduced empathy [6]. Due to their relevance, they were added to the Diagnostic and Statistical Manual of Mental Disorders (DSM-5) [7] as a subtyping specifier (“with limited prosocial emotion”) to the diagnosis of conduct disorder. Likewise, the International Classification of Diseases 11th revision (ICD-11) [8] included a similar specifier that can be applied to both conduct disorder (CD) and oppositional defiant disorder (ODD). Overall, studies have shown that CU traits delineate a group of children and adolescents with disruptive and aggressive behavior [9] that emerge earlier and are more severe and stable [10]. Moreover, CU traits are predictive of conduct problems and substance use during adolescence [11], and children and adolescents with CU traits are at greater risk for adult antisocial outcomes, including antisocial personality symptoms, delinquency, and arrests [12]. Finally, findings also suggested that children with externalizing behavioral problems and CU traits show a diminished response to traditional interventions [13], highlighting the need for a better understanding of these youths’ characteristics.

Several studies found associations between externalizing behavioral problems and intelligence measures, especially its verbal component; see, for instance, [14,15]. Few studies, instead, investigated the specific association between intelligence and CU traits.

Previous studies explored correlations between CU traits and intelligence measures. Javakhishvili and Vazsonyi [16] did not find relations between intelligence and CU traits in a longitudinal study with a large community sample of children. Loney et al. [17] found that children with CU traits and conduct problems reported only weaker nonverbal abilities. Allen et al. [18] found that CU traits were not related to poorer verbal and nonverbal abilities. Fontaine et al. [19] investigated the link between CU traits and intelligence in a wide community sample of children. They found that CU traits were negatively related to verbal and nonverbal abilities, but this relation did not remain significant after controlling for children’s externalizing problems. Finally, Sànchez de Ribera et al. [14] conducted a meta-analysis regarding the relations between psychopathy, antisocial behaviors, and intelligence in adults. They found a negative association between the affective facet of psychopathy (measured by CU traits in children) and intelligence.

Despite that extant studies provided some inconsistent evidence, there are plausible theoretical perspectives explaining why CU traits and IQ may be related. Intelligence has been strongly associated with emotional intelligence and theory of mind [20,21], which are central to the development of empathic skills and prosocial behavior. Studies have shown that cognitive abilities are connected to cognitive empathy and emotional awareness in childhood and preadolescence [20,22] and that the progressive improvement of cognitive abilities throughout the youths’ development fosters the increase in emotional awareness [20]. In this regard, the literature has consistently shown that empathy deficits are a core feature of CU traits [6,23] and that youths with high CU traits display severe difficulties in recognizing and understanding others’ emotions [24,25]; see also [26,27]. Research suggests that CU traits may be associated with specific cognitive deficits [6], which could at least partially account for the difficulties of children with CU traits in the empathy and emotional domains. More importantly, these specific cognitive deficits could represent distinct targets for intervention.

The current study aimed to explore the associations between parent-reported CU traits and intelligence, assessed with the WISC-IV in a sample of children referred for externalizing problems.

This study aimed to fill a gap in the extant literature, specifically previous studies focused on global intelligence scores or verbal vs. nonverbal/performance abilities assessed with short/abbreviated instruments. The use of more comprehensive measures of intelligence, such as the Wechsler Intelligence Scale for Children—4th Edition (WISC-IV) [28], which provides four different indexes, would guarantee a more nuanced evaluation of children’s intelligence and its relation to CU traits. Based on studies showing associations between empathy and intelligence and between externalizing problems and lower verbal ability, we hypothesized that CU traits would be associated with lower verbal comprehension scores.

## 2. Materials and Methods

### 2.1. Participants and Procedure

Participants were drawn from referrals to an outpatient service specialized in assessment and treatment for children and adolescents with externalizing problems located in Italy. All patients referred from January 2021 to December 2021 were enrolled in the study. Patients who received a diagnosis of autism spectrum disorder (*N* = 14) and/or a diagnosis of intellectual disability (*N* = 12) were excluded from the current study. The final sample included 149 children (84.60% males) aged 6–14 years (mean age = 9.63, *SD* = 2.09). Eighteen (12%) children were Africans, while all other participants were Caucasians. All participants were fluent in the Italian language. Forty-seven patients (31.5%) were diagnosed with attention-deficit hyperactivity disorder (ADHD), 19 patients (13.5%) with oppositional defiant disorder (ODD), and 83 patients (55.0%) with both ADHD and ODD.

The patients and their parents were invited to take part in the study. They were assured that participation in the study was voluntary and that refusal would not affect the clinical services they received in any way. After the parents signed a written informed consent and the child agreed to participate, a specialized psychologist conducted the assessment procedures. While the child was administered the intelligence tests, parents completed questionnaires assessing the child’s externalizing problems and CU traits. The study conformed to the Declaration of Helsinki, and the Ethical Committee of our hospital and the Regional Ethical Committee (Meyer Hospital, Florence) approved the study (N. 64/2019). All participants and their parents were required to sign a written informed consent before the beginning of the study.

### 2.2. Measures

*Externalizing Problems*. Externalizing problems were assessed with the Child Behavior Checklist (CBCL) [29]. Parents completed a checklist assessing behavioral problems and skills of their offspring. We used the Italian version of the CBCL [30]. Cronbach’s alpha was 0.76 in this sample.

*Callous–Unemotional Traits*. CU traits were assessed with six items of the Antisocial Process Screening Device (APSD) parent report. Parents rated each item on a three-point Likert scale (from *not at all true* to *definitely true*). The APSD has been frequently used in previous Italian studies; see, for instance, [31]. Cronbach’s alpha was 0.78 in this sample.

*Intelligence*. The Wechsler Intelligence Scale for Children—4th Edition (WISC-IV) [28] was used to assess children’s intelligence. For this study, we used the following indexes: verbal comprehension (VCI), perceptual reasoning (PRI), working memory (WMI), and processing speed (PSI). The WISC-IV has also been validated in an Italian sample [28].

### 2.3. Statistical Analysis

All the statistical tests were run on IBM SPSS Statistics for Windows, Version 26.0 (IBM Corp., Armonk, NY, USA) unless otherwise stated. We computed descriptive statistics and explored zero-order correlations among the study variables as preliminary analyses.

We then used linear regression to explore the associations between CU traits and the four WISC-IV indexes. The indexes were used as dependent variables, and CU traits were used as the independent variable. Alongside the WISC-IV indexes, we used gender, age, and externalizing problems as independent variables; based on the literature, we considered them to be possible confounding variables (e.g., [18]). Regression analysis was also used to explore whether CU traits and externalizing problems interacted in predicting the WISC-IV indexes. Variables were entered as in the previous models. All the variables that defined products were mean-centered prior to analysis.

Finally, we used independent sample t-tests to explore whether there were significant differences in the WISC-IV indexes between children with high CU traits and low externalizing problems and those with high CU traits and high externalizing problems. Participants were divided into low vs. high CU traits based on their APSD CU subscale scores. Those with a score ≥ 6 were included in the high-CU group (*N* = 41). Participants were then assigned to the low (*N* = 16) vs. high externalizing problems (*N* = 25) groups on the basis of a median split on the CBCL externalizing problems score. The median externalizing problems score within the high-CU group was 66. Forty-one children (27.52%) had high CU traits; sixteen (39.02%) of them had low externalizing problems, while the other 25 (60.90%) were included in the high externalizing problems group.

False discovery rate (FDR) correction of the *p*-values was applied across all regression models with the *p.adjust* function on R Statistics. A post hoc power analysis using the * Power 3.1.9 [32] was performed to estimate the power of our sample size. For an effect size settled at 0.10, given the mixed extant findings, and a level of significance for a *p*-value fixed at <0.05, our sample size had a power = 0.94 to test our hypothesis.

## 3. Results

Table 1 shows the descriptive statistics and zero-order correlations among the study variables. CU traits were negatively associated with VCI (*r* = −0.339, *p* ≤ 0.001, C.I. [−0.510, −0.240]) and PRI (*r* = −0.266, *p* = 0.001, C.I. [−0.429, −0.149]).

Linear regressions (Table 2) showed that CU traits were negatively associated only with VCI in our model (*adjusted R*^2^ = 0.245, *b* = −1.978, *β* = −0.336, *adjusted p* = 0.001, 95% C.I. [−2.936; −1.020]).

Further analyses showed that CU traits and externalizing problems did not interact in predicting the VCI (*b* = −0.077, *adj p* = 0.257, 95% C.I. [−0.186; 0.031]), PRI (*b* = 0.035, *adj p* = 0.617, 95% C.I. [−0.088; 0.158]), WMI (*b* = 0.009, *adj p* = 0.977, 95% C.I. [−0.113; 0.131]), or PSI (*b* = −0.043, *adj p* = 0.514, 95% C.I. [−0.175; 0.088]).

Finally, the independent sample t-tests showed that there were no significant differences between children with high CU traits and low externalizing problems and those with high CU traits and high externalizing problems in VCI (*t* = 0.730, *p* = 0.470), PRI (*t* = 0.729, *p* = 0.471), WMI (*t* = 0.534, *p* = 0.596), or PSI (*t* = 0.087, *p* = 0.931).

## 4. Discussion

The main objective of the present study was to identify possible relations between CU traits and cognitive abilities in a sample of children with severe behavioral problems. The results of this study showed a negative and significant association between the levels of CU traits and verbal abilities, measured with the verbal comprehension index of the WISC-IV. Unlike previous studies (e.g., [19]), this association remained significant even after controlling for externalizing problems. Interestingly, while we found that children with CU traits had poorer verbal abilities but preserved nonverbal abilities, Loney et al. [17] found the opposite result. Indeed, in their sample, those with high CU traits did not show a verbal deficit, but weaker nonverbal abilities. Despite similar sample compositions, the two studies showed some methodological differences that might account for the discrepancy in the findings. First, Loney et al. [17] used the WISC-R and WISC-III to assess children’s intellectual functioning, and those measures only provide a verbal scale IQ and a performance scale IQ instead of four different indexes. They also employed a different rating scale to assess CU traits, the Psychopathy Screening Device [33], and treated CU traits as a dichotomous (high vs. low) variable—as they tested group differences—while for our main analysis (linear regressions), it was used as a continuous variable.

Moreover, further analyses revealed that CU traits and externalizing problems did not interact in predicting the WISC-IV indexes and that there were no significant differences in the WISC-IV indexes between children with CU traits and high vs. low externalizing problems. This suggests that, in our sample, intelligence scores do not characterize those children with CU traits and a more severe clinical picture. Our results show that poorer verbal abilities are a unique characteristic of children with CU traits. This finding is in contrast with Fontaine et al. [19], who investigated the link between CU traits and cognitive abilities in children with typical development. They found that CU traits were related to lower verbal abilities, but not after controlling for externalizing problems.

Children with high levels of CU traits show a deficit of affective empathy, understood as the ability to share and “feel” the emotions of others while maintaining intact cognitive empathy, the ability to explicitly understand the emotional states and thoughts of other people from a purely cognitive point of view [34]. Verbal abilities are strongly related to empathy skills and emotion perception [35]. This might be particularly relevant for cognitive empathy, which also requires the ability to label emotions and properly communicate the understanding of others’ positions. The two facets of empathy work independently, but they also interact with each other, so we could speculate that specific treatment of children’s verbal abilities could improve cognitive empathy [21,36], which in turn could ameliorate affective empathy competencies. We can also hypothesize that lower verbal abilities are related to difficulties in emotional vocabulary and emotion understanding that promote the development of externalizing problems and CU behaviors [37]. Future experimental studies should test these hypotheses.

Previous studies indicated that youths with high CU traits can benefit from intensive interventions tailored to their unique emotional, cognitive characteristics [38,39]. As previously suggested by other authors [40], this work highlights the importance of taking into account verbal intelligence to buffer the development of CU traits. There are several programs developed to improve emotional intelligence, many of which are implemented in the school context; for a review, see [41].

The first innovative aspects of our study are the large sample of subjects and the significant negative correlation between verbal IQ and CU traits, which remained significant after also controlling for the levels of children’s externalizing behaviors. Another strong point is that the CU traits were measured through a parent report questionnaire, which, unlike the self-assessment one, returns a more objective measure of psychopathic traits [42]. Secondly, our study investigated intelligence competencies through the WISC-IV [28]. This allowed us to examine the relationships between CU traits and intelligence for both the verbal and nonverbal domains more nuancedly than previous studies, which focused on single domains of intelligence.

## 5. Limitations and Future Directions

Our findings must be viewed in light of some limitations. Firstly, no measures were used to assess variables that can influence the relationship we found (e.g., socioeconomic status, parenting, and language abilities), including contextual variables. Then, we used only parent-reported measures to assess externalizing problems and CU traits; the inclusion of teacher report instruments, too, would be highly informative. In addition, the cross-sectional nature of our work prevents us from inferring any causal link between the study variables. Finally, given the specific link between high levels of CU traits and lower cognitive verbal functioning detected, future research should also investigate the relationship between other dimensions of psychopathy, such as narcissism and impulsiveness, and cognitive functioning, in order to facilitate the understanding of possible etiological factors and to improve the treatment [24].

Overall, our results are in line with the extant literature, showing mixed evidence regarding the association between CU traits and cognitive functioning. This highlights the need for more research to achieve a better understanding of CU traits development and to find new intervention targets.

## Figures and Tables

**Table 1:** Descriptive statistics and zero-order correlations among the study variables.

	CU Traits	Age	Gender	Ext	VCI	PRI	WMI	PSI
CU traits	1							
Age	0.093	1						
Gender	0.064	0.177 *	1					
Ext	0.055	0.001	0.036	1				
VCI	−0.339 **	0.078	0.074	0.089	1			
PRI	−0.266 **	−0.034	0.061	0.006	0.399 **	1		
WMI	−0.045	0.102	0.026	0.043	0.275 **	0.377 **	1	
PSI	−0.085	0.144	−0.173 *	0.077	0.245 **	0.298 **	0.270 **	1
Mean	3.97	9.63	-	65.77	103.35	106.50	89.49	91.28
*SD*	2.30	2.09	-	9.42	13.38	15.75	13.89	15.49

Note. CU: callous–unemotional; Ext: externalizing problems; VCI: verbal comprehension index; PRI: perceptual reasoning index; WMI: working memory index; PSI: processing speed index. * *p* ≤ 0.05; ** *p* ≤ 0.001.

**Table 2:** Linear regression models with CU traits and covariates predicting VCI.

	*B*	*Beta*	*Adjusted p*	95% C.I. for *B*	
Age	0.018	0.033	0.686	−0.070	0.106
Gender	3.976	0.110	0.243	−1.914	9.866
Ext	0.151	0.107	0.243	−0.069	0.371
PRI	0.165	0.188	0.154	0.004	0.326
WMI	0.133	0.139	0.242	−0.031	0.296
PSI	0.097	0.110	0.243	−0.055	0.248
CU traits	−1.978	−0.336	0.001	−2.936	−1.020

Note. CU: callous–unemotional; Ext: externalizing problems; PRI: perceptual reasoning index; WMI: working memory index; PSI: processing speed index.

## Data Availability

Data are available from the corresponding author upon reasonable request.
